# Conditional knockout of the NSD2 gene in mouse intestinal epithelial cells inhibits colorectal cancer progression

**DOI:** 10.1002/ame2.12392

**Published:** 2024-02-23

**Authors:** Mengyuan Li, Hanxue Chen, Xingjiu Yang, Wenlong Zhang, Chengyan Ma, Qinghong Wang, Xinpei Wang, Ran Gao

**Affiliations:** ^1^ National Human Diseases Animal Model Resource Center Institute of Laboratory Animal Science, Chinese Academy of Medical Sciences and Peking Union Medical College Beijing China; ^2^ NHC Key Laboratory of Human Disease Comparative Medicine, Beijing Engineering Research Center for Experimental Animal Models of Human Critical Diseases Beijing China; ^3^ Beijing Engineering Research Center for Experimental Animal Models of Human Critical Diseases Beijing China; ^4^ Institute of Basic Medical Sciences, Chinese Academy of Medical Sciences, School of Basic Medicine Peking Union Medical College Beijing China

**Keywords:** colorectal cancer, NSD2^fl/fl^‐Vil1‐Cre mice, nuclear receptor‐binding SET domain 2

## Abstract

**Background:**

Nuclear receptor‐binding SET domain 2 (NSD2) is a histone methyltransferase, that catalyzes dimethylation of lysine 36 of histone 3 (H3K36me2) and is associated with active transcription of a series of genes. NSD2 is overexpressed in multiple types of solid human tumors and has been proven to be related to unfavorable prognosis in several types of tumors.

**Methods:**

We established a mouse model in which the NSD2 gene was conditionally knocked out in intestinal epithelial cells. We used azoxymethane and dextran sodium sulfate to chemically induce murine colorectal cancer. The development of colorectal tumors were investigated using post‐necropsy quantification, immunohistochemistry, and enzyme‐linked immunosorbent assay (ELISA).

**Results:**

Compared with wild‐type (WT) control mice, NSD2^fl/fl^‐Vil1‐Cre mice exhibited significantly decreased tumor numbers, histopathological changes, and cytokine expression in colorectal tumors.

**Conclusions:**

Conditional knockout of NSD2 in intestinal epithelial cells significantly inhibits colorectal cancer progression.

## Graphical abstract

In this study, we established a mouse strain in which the NSD2 gene was conditionally knocked out in intestinal epithelial cells. Afterward, azoxymethane (AOM) and dextran sodium sulfate (DSS) were used to initiate colorectal cancer in mice. Our results showed that conditional knockout of NSD2 in intestinal epithelial cells inhibited the progression of colorectal cancer in mice.

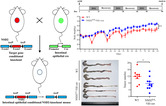

## 1 INTRODUCTION

Nuclear receptor‐binding SET domain 2 (NSD2) is also known as Wolf–Hirschhorn syndrome candidate 1 or multiple myeloma SET domain and belongs to the nuclear receptor‐binding SET domain (NSD) family of histone 3 lysine 36 (H3K36) methyltransferases.^1^ NSD family methyltransferases have similar structures and are involved in maintaining chromatin integrity, and regulating a variety of cellular biological processes, such as proliferation, apoptosis, DNA damage repair, and epithelial–mesenchymal transition (EMT).^2^

A large number of studies have found that NSD2 is related to various cellular processes that can induce tumorigenesis. Studies have also shown overexpression of NSD2 in multifarious solid tumors, including lung cancer, prostate cancer, colorectal cancer, cervical cancer, breast cancer, and osteosarcoma.^3^, ^4^, ^5^, ^6^, ^7^, ^8^, ^9^, ^10^, ^11^ This observation suggests that NSD2 potentially plays a critical role in tumor cell growth, proliferation, migration, invasion, and EMT.^2^ Other studies have reported that NSD2 expression is dynamically regulated during cell‐cycle progression and promotes normal DNA replication.^12^ NSD2 regulates Wnt‐ and NF‐kB‐related pathways to support tumor growth and survival.^13^, ^14^ NSD2 mediates the proliferation and apoptosis of tumor cells by regulating the expression of tumor‐related cytokines, such as tumor necrosis factor alpha (TNF‐α), interleukin 6 (IL‐6), IL‐8, and vascular endothelial growth factor A, or by reducing p53 stability.^14^, ^15^ Song et al. found that NSD2 also promotes tumor angiogenesis by methylating STAT3 and activating the STAT3 pathway.^16^

Colorectal cancer is a common heterogeneous tumor with the fourth‐highest morbidity and the second‐highest mortality among all malignancies. Currently, colorectal cancer remains deadly for many patients.^17^ An in‐depth understanding of the pathological mechanisms of colorectal cancer progression is crucial for the early diagnosis and treatment of this deadly disease.^18^ In this study, we established a mouse strain in which the NSD2 gene was conditionally knocked out in intestinal epithelial cells. Then, azoxymethane (AOM) and dextran sodium sulfate (DSS) were used to initiate colorectal cancer in mice.^19^, ^20^, ^21^ We found that conditional knockout of NSD2 in intestinal epithelial cells inhibited the progression of colorectal cancer in mice. This shows that NSD2 plays a critical role in the process of colorectal cancer progression and could be a potential therapeutic target for colorectal cancer.

## 2 MATERIALS AND METHODS

### 2.1 Animals

NSD2‐floxp (NSD2^fl/fl^) mice and Vil1‐Cre mice were purchased from Beijing Viewsolid Biotechnology Co. Ltd. All animal experiments were approved by the Animal Experiments Committee of the Chinese Academy of Medical Sciences (IACUC: GR23001). NSD2^fl/fl^ mice were crossed with Vil1‐Cre mice to produce intestinal epithelial cell‐specific NSD2 knockout mice (NSD2^fl/fl^‐Vil1‐Cre mice); 8‐ to 10‐week‐old male NSD2^fl/fl^‐Vil1‐Cre mice and C57BL/6J wild‐type (WT) mice were divided into two groups for subsequent experiments. The genotype of the transgenic mice was confirmed using polymerase chain reaction (PCR). The efficiency of NSD2 knockout was determined using Western blotting.

### 2.2 PCR assay

Total DNA was extracted from the tail tips of mice using an EasyPure Genomic DNA Kit (EE101‐12, TransGen Biotech) following the manufacturer's instructions. The total reaction volume used for PCR was 20 μL: genomic DNA, 2 μL; forward primer (10 μmol/L), 1 μL; reverse primer (10 μmol/L), 1 μL; Taq PCR mix (RR901A, Takara), 10 μL; and ddH_2_O, 6 μL. The thermal cycling conditions were as follows: 95°C for 3 min; 35 cycles at 95°C for 30 s, 60°C for 30 s, and 72°C for 40 s; 72°C for 10 min; and holding at 4°C.The primers used for PCR are presented in Table 1. PCR products were analyzed using electrophoresis in a 1.5% agarose gel.

**TABLE 1:** Primer sequences for PCR.

Names	Primer sequence (5′–3′)
NSD2‐LOXP‐FP	TTCTGCTGAAGTCTTGTCTT
NSD2‐LOXP‐RP	CTTGGTGTTGTTTGTGCTTA
Vil1‐Cre‐F	GTGTGGGACAGAGAACAAACCG
Vil1‐Cre‐R	TGCGAACCTCATCACTCGTTGC

Abbreviation: PCR, polymerase chain reaction.

### 2.3 Western blotting

Colorectal tissues and colorectal cancer cells were collected and homogenized in RIPA lysis buffer (R0010, Solarbio). Lysates containing equal amounts of cellular protein were loaded and separated on 12.5% sodium dodecyl sulfate‐polyacrylamide gel electrophoresis gels, and proteins were electrotransferred to polyvinylidene fluoride (PVDF) membranes. After blocking, the PVDF membranes were incubated with anti‐NSD2 (ab75359, Abcam), anti‐Histone H3 (dimethylated at K36) (ab176921, Abcam), anti‐Histone H3 (ab1791, Abcam), anti‐phospho‐Akt (Ser473) (9271S, CST), anti‐Akt (9272S, CST), anti‐GAPDH (AG019, Beyotime), and anti‐β‐actin (ab49900, Abcam) antibodies overnight at 4°C. After the PVDF membranes were washed thrice with TBST buffer, they were incubated in horseradish peroxidase (HRP)–conjugated IgG (ZDR‐5307, ZSGB‐Bio) for 1 h at room temperature. Finally, the bands on the PVDF membranes were detected using a Bio‐Rad detection system.

### 2.4 Colorectal cancer model

Colorectal cancer was chemically induced in mice by intraperitoneal injection of 12 mg/kg of AOM (A5486, Sigma), which is a colon organotropic carcinogen, dissolved in 0.9% sodium chloride. After 5 days of rest, colitis was induced in mice by administering 2.5% DSS (9011‐18‐1, Merck) dissolved in drinking water for 5 days. Then, regular water was provided for 2 weeks. Next, two rounds of 2.5% DSS were administered (Days 25–29 and 44–48). Finally, the mice in these two groups were killed and analyzed on Day 90.

### 2.5 Immunohistochemistry

Colorectal tissues were collected and fixed in 10% formaldehyde solution for 36–48 h. Then, dehydration, paraffin embedding, and tissue sectioning were performed. The sections were deparaffinized in xylene, rehydrated in a graded ethanol series, boiled in antigen retrieval solution for 15 min, and incubated with 3% H_2_O_2_ to quench endogenous peroxidase activity. Next, the sections were blocked for 1 h and incubated with anti‐CD31 (ab182981, Abcam) and anti‐Ki67 (ab16667, Abcam) antibodies overnight at 4°C in a humidified chamber. Then, the sections were incubated with a HRP‐conjugated secondary antibody for 1 h. Prepared diaminobenzidine was added to the sections, and nuclei were counterstained with Mayer's hematoxylin after washing with phosphate‐buffered saline. Finally, the sections were dehydrated, covered with a coverslip, and visualized using a Leica microscope.

### 2.6 Enzyme‐linked immunosorbent assay

Mouse blood was collected on Day 90 and centrifuged to obtain serum. The concentrations of IL‐6, TNF‐α, and transforming growth factor‐β1 (TGF‐β1) in serum were measured using Quantikine ELISA kits (M6000B, MTA00B, DY1679, R&D Systems) following the manufacturer's instructions. The absorbance rate of each well at 450 nm was determined using a microplate reader (Bio‐Rad, USA).

### 2.7 Cell lines and transfection

The colorectal cancer cell line MC38 and the NSD2‐knockdown vector pll3.7‐shNSD2 were obtained from the Institute of Basic Medical Sciences, Chinese Academy of Medical Sciences. MC38 cells were transfected with Lipofectamine 3000 (L3000001, Invitrogen) and harvested after 48 h for further analysis.

### 2.8 Cell proliferation assay

Cells were seeded into 96‐well plates at a density of 3 × 10^3^ cells per well. On the day of seeding, one group of cells was incubated with CCK‐8 reagent (1:100 dilution) at 37°C. After 3 h, the absorbance rate of the culture supernatants at 450 nm was measured using a microplate reader, and one group of culture supernatants was then measured every 24 h until the 96‐h time point.

### 2.9 Colony formation assay

Cells were seeded into six‐well plates at a density of 1 × 10^3^ cells per well and cultured at 37°C. When the number of cells in a single colony was greater than 50, the cells were fixed with 4% paraformaldehyde and then stained with 0.1% crystal violet. After the cells were washed, the number of colonies were determined using a microscope.

### 2.10 Statistical analysis

GraphPad Prism software, version 8.0, was used for statistical analysis. All the data are expressed as means ± standard deviation. Statistical differences between the two groups were determined using the independent samples *t*‐test, whereas ANOVA (analysis of variance) was used for comparisons among multiple groups. Statistical significance is reported as *p < 0.05, **p < 0.01, or ***p < 0.001.

## 3 RESULTS

### 3.1 Establishment of NSD2^fl^ ^/^ ^fl^‐Vil1‐Cre mice

**FIGURE 1:**
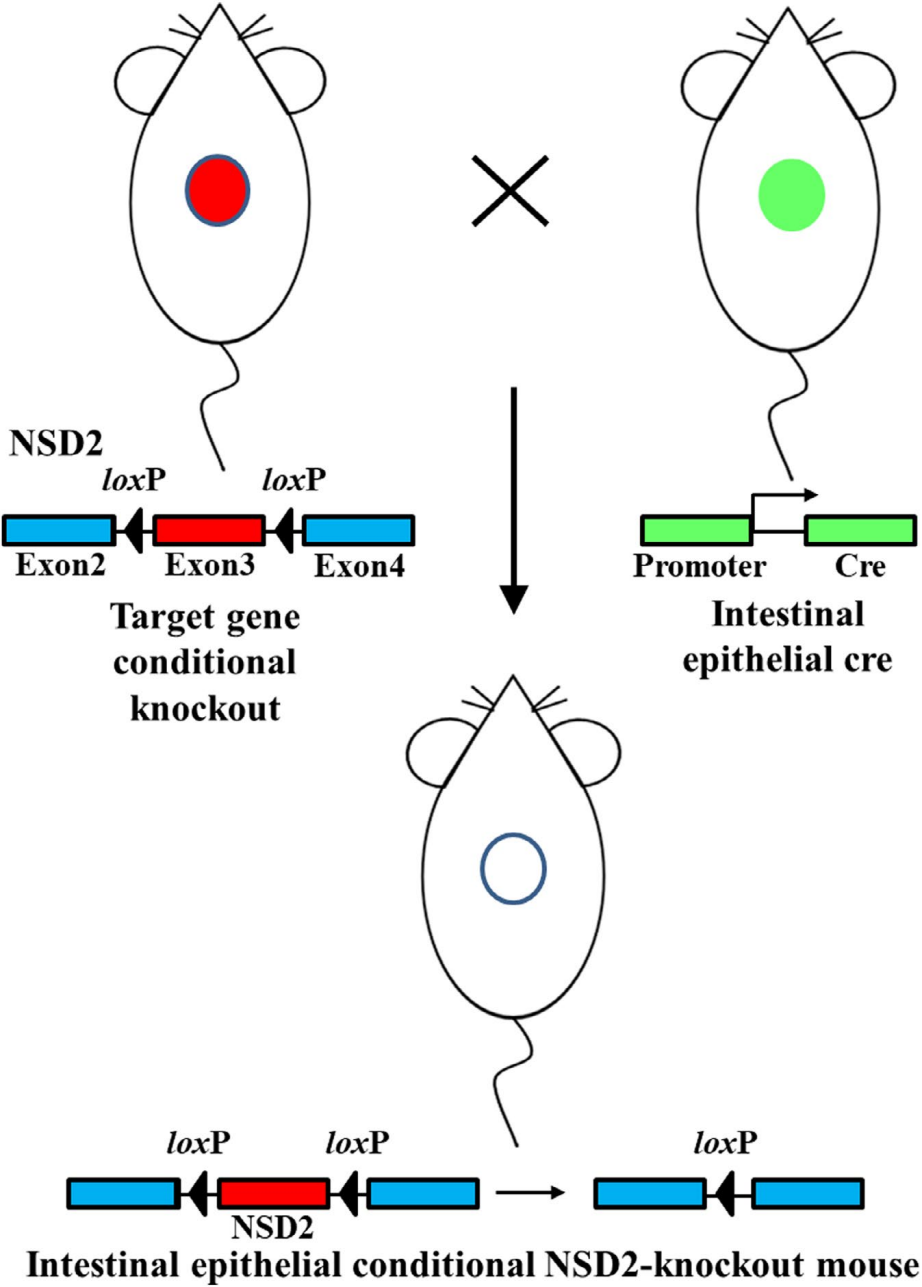
Establishment of NSD2^fl/fl^‐Vil1‐Cre mice.

According to the genome structure and conserved functional domain in the encoded protein, NSD2 has multiple transcripts. Of these transcripts, exon 3, which encodes a 167‐bp sequence, is the common exon located in the sequence encoding the conserved functional domain of the protein, that is, Msh6_like. Conditional deletion of exon 3 may disrupt the Msh6_like sequence and form a new messenger RNA (mRNA) transcript with frameshift mutations, which leads to protein inactivation.^1^, ^2^ Therefore, a FloxP site was inserted upstream and downstream of exon 3 to knock out exon 3 and accomplish conditional knockout of the NSD2 gene. Moreover, two strains of mice need to mate for conditional knockout of target genes. After mating, exon 3 of the NSD2 gene, sandwiched between the two FloxP loci, was deleted in intestinal epithelial cells with specific expression of cyclization recombination enzyme (Cre). This resulted in inactivation and conditional knockout of NSD2 (Figure 1).

### 3.2 Identification of NSD2^fl^ ^/^ ^fl^‐Vil1‐Cre mice

**FIGURE 2:**
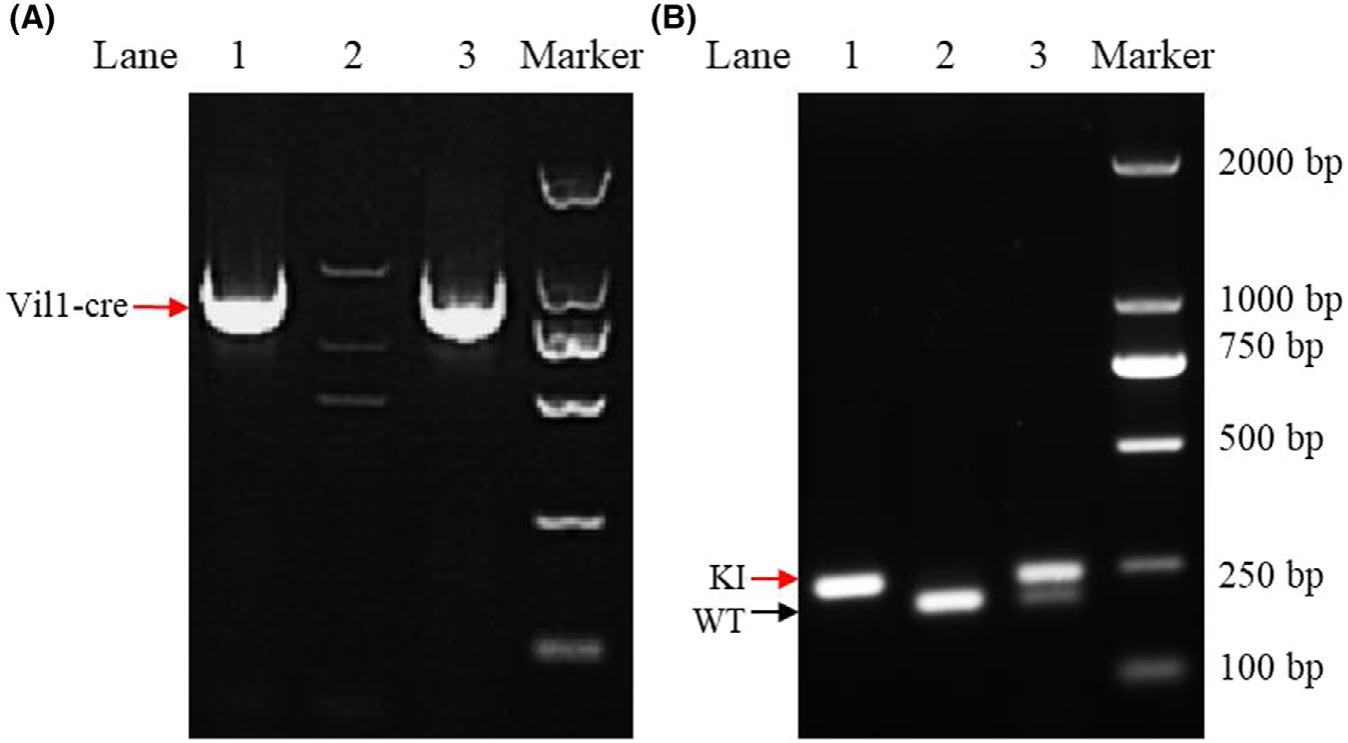
Results of mouse genotyping by PCR. (A) 1, 3. Vil1‐Cre‐positive mice; 2. WT mice. (B) 1. NSD2^fl/fl^ homozygous mice; 2. WT mice; 3. NSD2^fl/+^ heterozygous mice.

**FIGURE 3:**
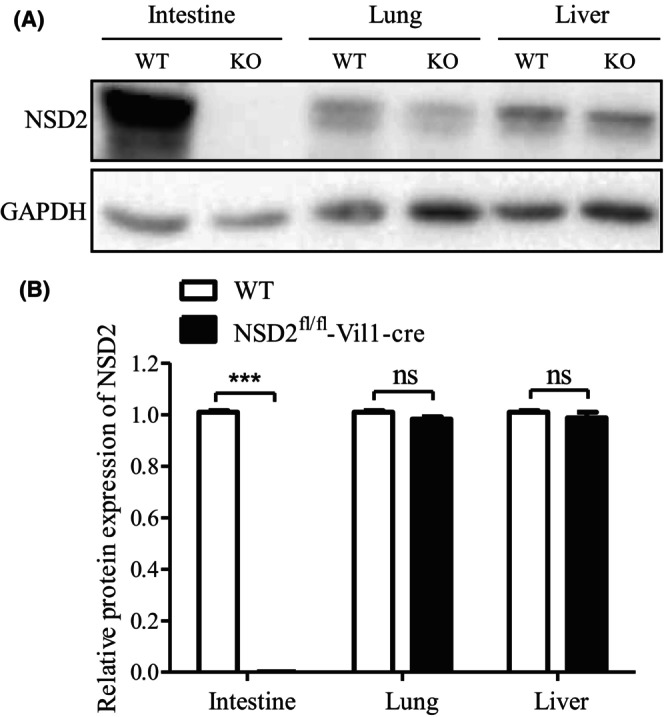
NSD2 is conditionally knocked out in the mouse intestine, but not in other tissues. The results are presented as the means ± SDs; *n* = 3, ***p < 0.001.

NSD2 expression was analyzed using PCR and Western blotting. Figure 2A shows that lane 2 exhibits no band. The band size in lanes 1 and 3 was 921 bp; thus, mice 1 and 3 are Vil‐cre mice. Figure 2B shows that the band size in lane 2 was 209 bp. The band sizes in lane 3 were 209 and 243 bp. The band size in lane 1 was 243 bp. Therefore, mouse 2 is a WT mouse, mouse 3 is an NSD2^fl/+^‐Vil1‐Cre heterozygous mouse, and mouse 1 is an NSD2^fl/fl^‐Vil1‐Cre homozygous mouse. As shown in Figure 3, Western blot results indicated that NSD2 was conditionally knocked out in the mouse intestine (*p* < 0.001) but not in other tissues, such as the lung or liver.

### 3.3 Phenotypic analysis of NSD2^fl^ ^/^ ^fl^‐Vil1‐Cre mice

**FIGURE 4:**
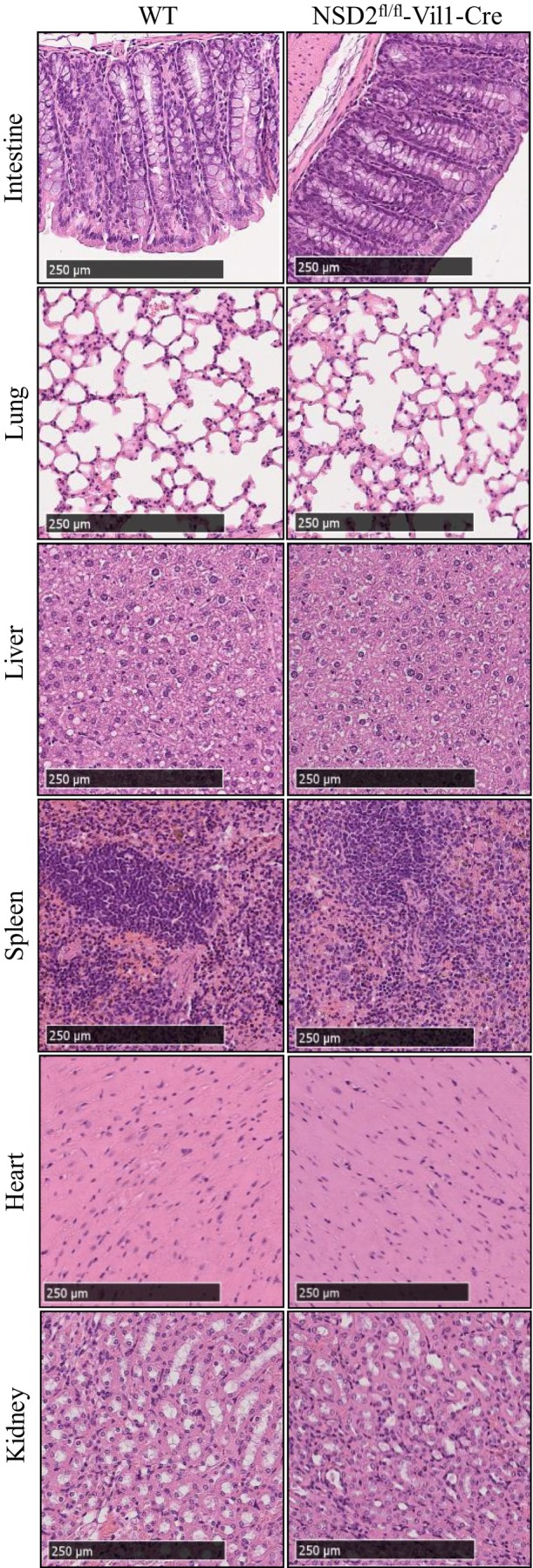
There are no abnormalities in the major organs of NSD2^fl/fl^‐Vil1‐Cre mice compared with those of WT mice.

Twelve‐week‐old male mice were used in this experiment (Figure 4). There was no significant difference in appearance between intestinal epithelial conditional NSD2‐knockout mice and WT mice on the C57BL/6J genetic background. Compared with WT mice, NSD2^fl/fl^‐Vil1‐Cre mice showed no abnormalities in the major organs on staining with hematoxylin–eosin. None of the mice spontaneously developed tumors.

### 3.4 Knockout of NSD2 inhibits colorectal tumor growth

**FIGURE 5:**
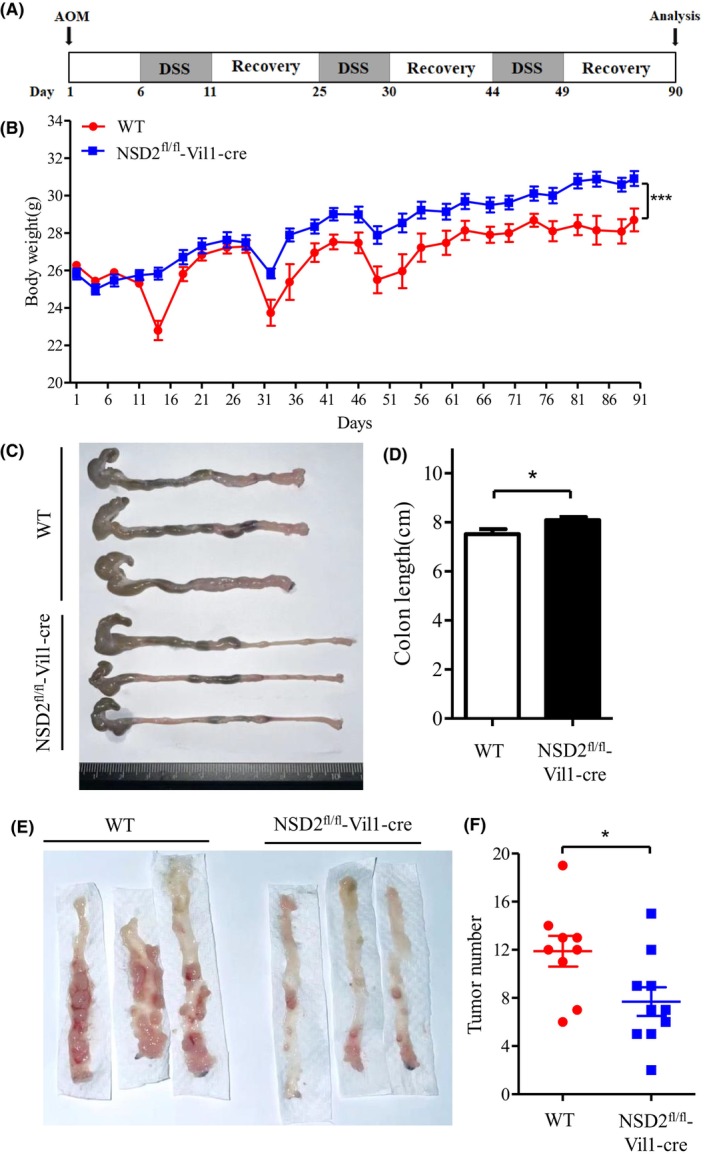
The growth of colorectal tumors are inhibited in NSD2^fl/fl^‐Vil1‐Cre mice. (A) WT and NSD2^fl/fl^‐Vil1‐Cre mice were injected with azoxymethane (AOM) intraperitoneally on Day 1. Then, the mice were treated with three rounds of 2.5% DSS administration on Days 6–11, 25–29 and 44–48, and allowed to recover with normal drinking water provided until Day 90. (B) The mean changes in body weight were measured every 3 days until Day 90. (C,D) Colon lengths were measured on Day 90. (E,F) Tumor numbers in the colon tissues were determined on Day 90. The results are presented as the means ± SDs; WT (*n* = 9), NSD2^fl/fl^‐Vil1‐Cre (*n* = 10), *p < 0.05, ***p < 0.001.

Figure 5A shows the time course of the experiment. All mice were injected with AOM intraperitoneally on Day 1. Then, the mice were subjected to three rounds of 2.5% DSS administration on Days 6–11, 25–29, and 44–48 and allowed to recover with normal drinking water provided until Day 90. Compared with that of WT mice, the body weight of NSD2^fl/fl^‐Vil1‐Cre mice increased significantly during the experiment (Figure 5B). Additionally, NSD2^fl/fl^‐Vil1‐Cre mice showed fewer symptoms of colitis, including unformed stool, rectal bleeding, and decreased colon length (Figure 5C,D). In the NSD2^fl/fl^‐Vil1‐Cre mouse group, the number of tumors in the colon was significantly decreased (Figure 5E,F).

### 3.5 Knockout of NSD2 inhibits tumor cell proliferation and tumor angiogenesis

**FIGURE 6:**
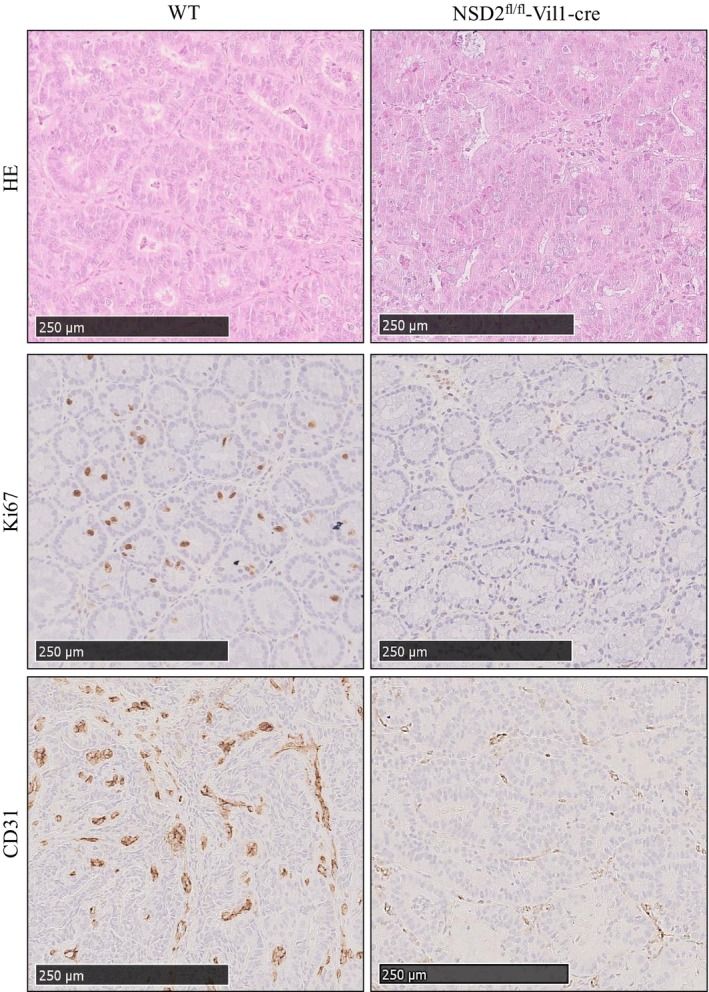
Knockout of NSD2 inhibits tumor cell proliferation and tumor angiogenesis.

It has been reported that upregulation of NSD2 is positively correlated with tumor cell proliferation and tumor angiogenesis.^4^, ^5^, ^11^ We evaluated the expression of Ki67 and CD31 considering that NSD2 knockout significantly inhibited tumor growth (Figure 5E,F). The immunohistochemical staining results showed that knockout of NSD2 inhibited the expression of Ki67 and CD31 (Figure 6). This inhibition of Ki67 and CD31 suggested that tumor proliferation and angiogenesis decreased with NSD2 knockout in the colon.

### 3.6 Knockout of NSD2 inhibits the expression of inflammatory cytokines

**FIGURE 7:**
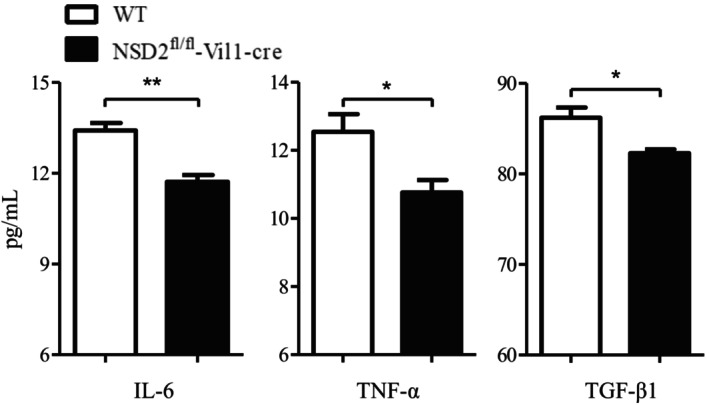
Knockout of NSD2 inhibits the expression of inflammatory cytokines. The results are presented as the means ± SDs; WT (*n* = 9), NSD2^fl/fl^‐Vil1‐Cre (*n* = 10), *p < 0.05, **p < 0.01.

Upregulation of NSD2 is correlated with increased expression levels of IL‐6, TNF‐α, and TGF‐β1, which can affect the development of other types of solid tumors.^14^, ^22^ Therefore, we measured the concentrations of IL‐6, TNF‐α, and TGF‐β1 in mouse serum. Our results showed that the concentrations of IL‐6, TNF‐α, and TGF‐β1 in the serum of NSD2^fl/fl^‐Vil1‐Cre mice with colorectal cancer were much lower than those in the serum of WT mice (Figure 7).

### 3.7 Knockdown of NSD2 inhibits H3K36 methylation and Akt activation in vitro

**FIGURE 8:**
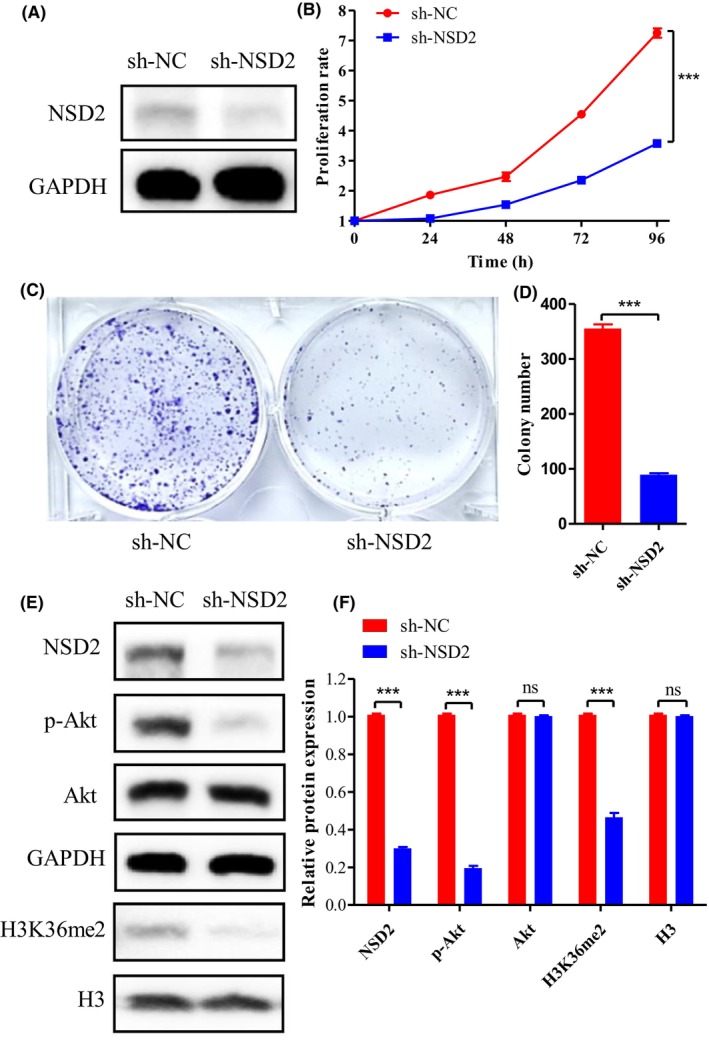
Knockdown of NSD2 inhibits H3K36 methylation and Akt activation in vitro. (A) Western blotting was used to evaluate the expression of the NSD2 protein in MC38 cells after NSD2 knockdown. (B–D) CCK‐8 and colony formation assays were performed to measure the proliferation of MC38 cells. (E,F) Western blotting was performed to measure the expression of proteins in associated signaling pathways. The results are presented as the means ± SDs; *n* = 3, ***p < 0.001.

We transfected the pll3.7‐shNSD2 vector into the mouse colorectal cancer cell line MC38 to induce NSD2 knockdown. Figure 8A shows that the expression of the NSD2 protein in MC38 cells was downregulated after transfection. CCK‐8 and colony formation assays showed that the proliferation ability of NSD2‐knockdown cells were significantly decreased (Figure 8B–D). Western blot analysis revealed that the H3K36me2 level was significantly decreased after NSD2 knockdown. Moreover, Akt phosphorylation was inhibited (Figure 8E,F).

## 4 DISCUSSION

The Gene Expression Profiling Interactive Analysis (http://gepia.cancer‐pku.cn/) database shows that NSD2 mRNA levels in colon and rectal cancer tissues are significantly higher than those in normal tissues.^11^ These results suggest that NSD2 is a crucial oncogene in colorectal cancer. In this study, we established a mouse model in which the NSD2 gene was conditionally knocked out in intestinal epithelial cells. We used AOM/DSS treatment to induce colorectal cancer in mice. The AOM/DSS model is an efficient, reproducible, and relatively inexpensive model of colitis‐associated cancer. It has a relatively short timeline and is an accurate model of colitis‐associated cancer.^20^, ^23^, ^24^ In this experiment, the body weight of NSD2^fl/fl^‐Vil1‐Cre mice was significantly increased compared to that of WT mice. NSD2^fl/fl^‐Vil1‐Cre mice also exhibited fewer colitis symptoms and fewer tumors. Thus, these results indicate that conditional knockout of NSD2 in the intestine can inhibit the progression of colorectal cancer.

D'Afonseca et al. showed that NSD2 expression has a strong correlation (*r* ^2^ = 0.6881) with Ki67 mRNA expression, which suggests that NSD2 can play a similar role or participate in a similar proliferation pathway as Ki67.^5^ Song et al. showed that NSD2 interacts with STAT3 to methylate it and can change its phosphorylation level. NSD2 catalyzes STAT3 methylation, which contributes to the activation of the STAT3 pathway and tumor angiogenesis.^16^ Our results showed that knockout of NSD2 inhibited the expression of Ki67 and CD31 in colorectal tumor tissues, suggesting that overexpression of NSD2 can promote the proliferation and angiogenesis of colorectal tumors. It has been reported that upregulation of NSD2 is correlated with increased expression of IL‐6 and TNF‐α via NF‐κB in prostate cancer.^14^ EMT induced by TGF‐β1 promotes the invasion and migration of cervical cancer cells. NSD2 knockout suppresses metastasis in cervical cancer by inhibiting TGF‐β/TGF‐βRI/SMAD signaling.^22^ The aforementioned cytokines play important roles in colitis‐associated cancer.^19^, ^21^, ^25^ We found that knockout of NSD2 significantly reduced the concentrations of IL‐6, TNF‐α, and TGF‐β1 in the serum of mice with colorectal cancer. NSD2^fl/fl^‐Vil1‐Cre mice showed fewer symptoms of colitis, including unformed stool, rectal bleeding, and decreased colon length. Therefore, knockout of NSD2 can inhibit the progression of colorectal cancer by reducing the levels of inflammatory cytokines.

D'Afonseca et al. found that NSD2 was a marker of poor prognosis in colorectal cancer patients through biological data mining.^5^ Zhao et al. found that NSD2 silencing led to the inhibition of Akt activation in primary colorectal cancer cells. In the related experiments, colorectal cancer cells were subcutaneously injected into the flanks of nude mice to establish tumor models.^11^ Chen et al. found that NSD2 circular RNA, by acting as a sponge of miR‐199b‐5p, promoted colorectal cancer invasion and metastasis by upregulating DDR1 and JAG1. In the related experiments, C57BL/6J mice were used to establish a model of colorectal cancer liver metastasis, and BALB/c nude mice were used to establish a subcutaneous colorectal tumor model and a model of colorectal cancer lung metastasis.^4^ All of these tumor models were established by injecting colorectal cancer cells, in contrast to our approach. The model of spontaneous colorectal tumors induced by AOM/DSS can more realistically simulate the occurrence and development of human tumors.^20^ Moreover, the aforementioned studies confirmed that NSD2 silencing inhibited the development of colorectal cancer in both C57BL/6J mice and nude mice and that overexpression of NSD2 promoted the development of colorectal cancer.^4^, ^11^, ^26^ This result suggested that the intact immune system does not affect the biological function of NSD2.

Our studies confirmed that NSD2 knockdown inhibited the proliferation of MC38 cells in vitro. The level of H3K36me2 in MC38 cells were significantly inhibited after NSD2 knockdown. Because H3K36me2 controls gene expression and affects DNA repair,^2^ NSD2‐mediated H3K36me2 is essential for the transcriptional activation and expression of multiple oncogenes to support the development of colorectal cancer. Previous studies have shown that NSD2 plays an important role in Akt activation.^7^, ^11^ Our results showed that the level of phosphorylated AKT decreased after NSD2 knockdown, although the level of total AKT was not altered. Therefore, NSD2 regulates the AKT signaling pathway by mediating the H3K36me2 modification of gene loci in colorectal cancer.

In conclusion, we established a model of colorectal cancer in which intestinal epithelial cells were subjected to conditional NSD2 knockout. Not only is this model a powerful tool to study the pathogenesis of colorectal cancer, but also our findings indicate that NSD2 may serve as a target for new therapeutic strategies for colorectal cancer. NSD2 may play a critical role in the development and prognosis of human cancers and may represent a new therapeutic target for human solid tumors.

## AUTHOR CONTRIBUTIONS

Ran Gao and Mengyuan Li designed the experiment. Mengyuan Li, Hanxue Chen, Xingjiu Yang, and Wenlong Zhang performed the experiment. Chengyan MA, Qinghong Wang, and Xinpei Wang carried out data analysis. Ran Gao and Mengyuan Li prepared the manuscript. All authors have read and approved the final manuscript.

## CONFLICT OF INTEREST STATEMENT

The authors declare no competing interests. Ran Gao is an editorial board member of *AMEM* and a coauthor of this article. To minimize bias, she was excluded from all editorial decision making related to the acceptance of this article for publication.
